# A novel AlGaN/GaN heterostructure field-effect transistor based on open-gate technology

**DOI:** 10.1038/s41598-021-01917-9

**Published:** 2021-11-17

**Authors:** Yang Liu, Yuanjie Lv, Shuoshuo Guo, Zhengfang Luan, Aijie Cheng, Zhaojun Lin, Yongxiong Yang, Guangyuan Jiang, Yan Zhou

**Affiliations:** 1 School of Microelectronics, Institute of Novel Semiconductors, Shandong University, Jinan, 250101 China; 2 National Key Laboratory of Application Specific Integrated Circuit (ASIC), Hebei Semiconductor Research Institute, Shijiazhuang, 050051 China; 3 School of Mathematics, Shandong University, Jinan, 250100 China

**Keywords:** Electronic devices, Electronic and spintronic devices

## Abstract

In this study, a novel AlGaN/GaN heterostructure field-effect transistor based on open-gate technology was fabricated. Sample transistors of different structures and sizes were constructed. Through measurements, it was found that by changing the width of the opening, the threshold voltage of the device could be easily modulated across a larger range. The open-gate device had two working modes with different transconductance. When the gate-source voltage *V*_GS_ ≤  − 4.5 V, only the open region was conductive, and a new working mechanism modulated the channel current. Corresponding theoretical analysis and calculations showed that its saturation mechanism was related to a virtual gate formed by electron injection onto the surface. Also, the gate-source voltage modulated the open channel current by changing the channel electron mobility through polarization Coulomb field scattering. When used as class-A voltage amplifiers, open-gate devices can achieve effective voltage amplification with very low power consumption.

## Introduction

The AlGaN/GaN heterostructure field-effect transistor (HFET), a type of wide-bandgap semiconductor electronic device, has the advantages of high breakdown voltage and high electron mobility, which has led to it being widely used in high frequency and high power applications^1–6^. Whether for switching devices or RF power devices, the threshold voltage (*V*_th_) is a very important parameter. However, the common methods to change the *V*_th_ of AlGaN/GaN HFETs are relatively complex. It is usually necessary to change the epitaxial structure of the material or introduce new process steps, such as changing the thickness, doping concentration or Al composition of the barrier layer, plasma treatment of the material surface, growing an insulating layer with a certain thickness above the barrier layer, etc^7–12^. It is extremely difficult to change the *V*_th_ of the device across a large range by simply adjusting the device structure.

Additionally, polarization Coulomb field (PCF) scattering is an important scattering mechanism in AlGaN/GaN HFETs. It has an important influence on the gate region electron mobility, gate-source resistance, extrinsic transconductance, linearity and other characteristics of the devices^13–17^. To a certain extent, *V*_th_ can reflect the voltage range in which the gate-source voltage (*V*_GS_) can effectively modulate the drain-source current (*I*_DS_), and PCF scattering is related to both *V*_GS_ and *I*_DS_. Therefore, it should be entirely possible to design a new device structure using the mechanism of PCF scattering to achieve large-scale modulation of *V*_th_ in AlGaN/GaN HFETs.

In this paper, a novel AlGaN/GaN HFET which had an open gate structure and a new working mechanism was designed. The open-gate device had two working modes with different modulation abilities. Under the condition that the gate region two-dimensional electron gas (2DEG) was depleted, the open region electron mobility could be modulated by PCF scattering, to enable the modulation of *I*_DS_ by *V*_GS_. After the introduction of the open gate structure, a simple method of changing the width of the gate opening could be used to modulate *V*_th_ across a larger range. As class-A voltage amplifiers, open-gate devices can achieve effective voltage amplification with very low power consumption. The open-gate device in this paper had two independent gates, which would increase the complexity of device packaging, but also provided an extra degree of freedom for its application in the circuit. In addition, a new device structure can be designed to connect two gates to the same pad to avoid complex packaging.

## Results and discussion

### Analysis of device characteristics

**Figure 1:**
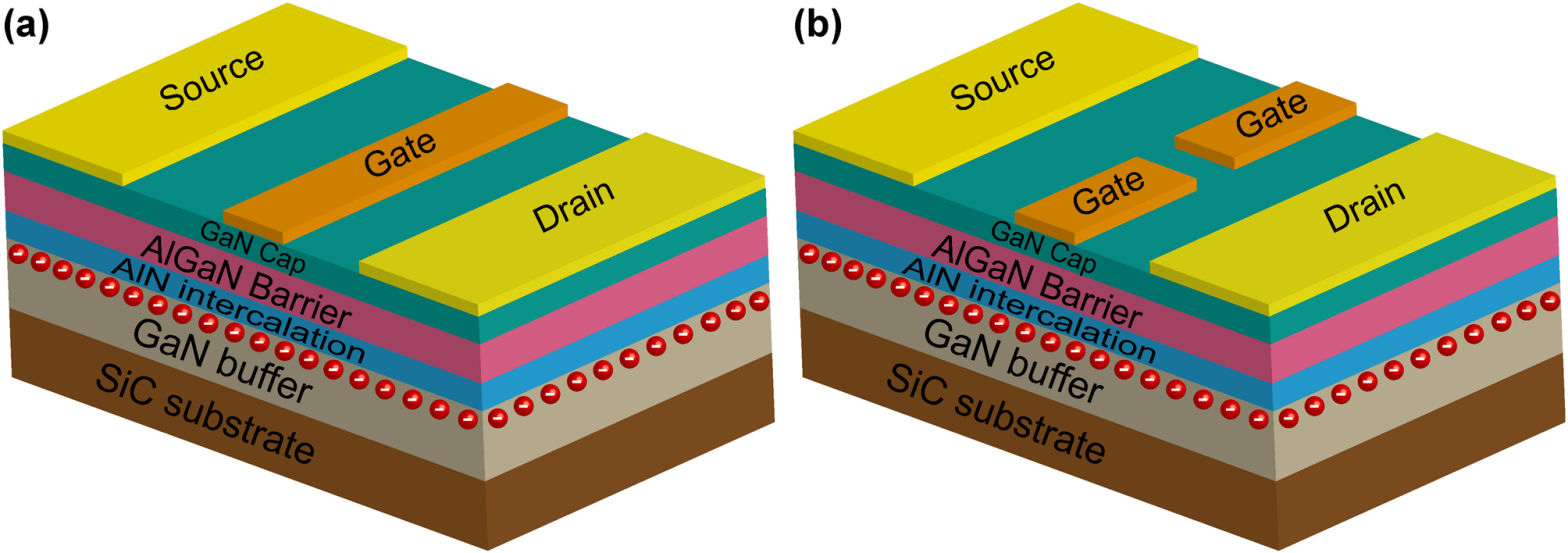
Schematic diagram of the structure of (**a**) normal device and (**b**) open-gate device. The figure was generated by Adobe Illustrate CC 2019 (https://www.adobe.com/cn/products/illustrator.html).

Four AlGaN/GaN HFETs with different structures and sizes were prepared, and the structural difference between normal device and open-gate device is shown in Fig. 1. For these four samples, the gate length (*L*_G_) was 40 μm, the total channel width (*W*) was 100 μm, and the gate-source distance (*L*_GS_) and gate-drain distance (*L*_GD_) were both 6 μm. Sample 1 was a normal device, with an unopened gate. Samples 2, 3, and 4 were all devices with an opening in the middle of the gate, and the opening widths (*W*_O_) were 3 μm, 4 μm, and 5 μm, respectively. The large device size is related to the working mechanism of the open-gate device, which will be discussed in detail later.

**Figure 2:**
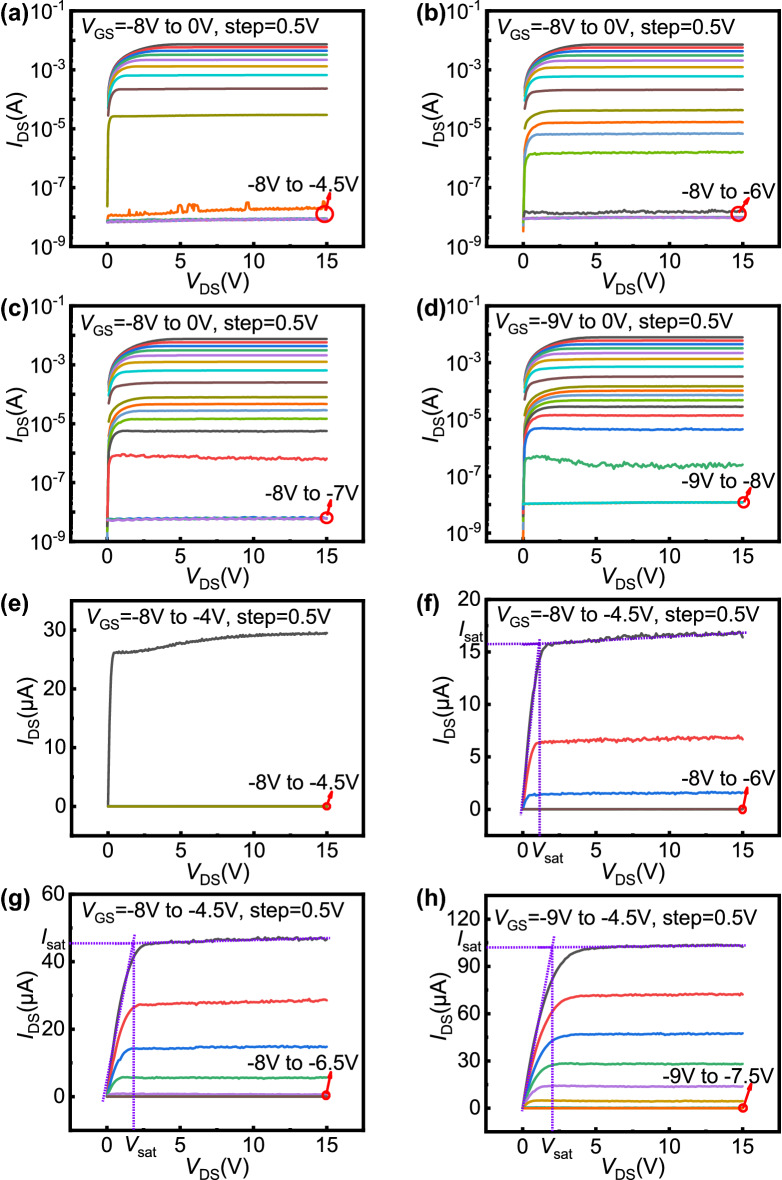
The measured output characteristics with logarithmic y-axis: (**a**) sample 1, (**b**) sample 2, (**c**) sample 3, and (**d**) sample 4. The measured output characteristics with standard y-axis at a specific range of gate bias: (**e**) sample 1, (**f**) sample 2, (**g**) sample 3, and (**h**) sample 4.

The output characteristics with logarithmic y-axis and standard y-axis of the four samples are shown in Fig. 2. It can be clearly seen that for these four samples, *V*_GS_ had a good ability to modulate *I*_DS_. Even for open-gate samples, *I*_DS_ could still be modulated by *V*_GS_ until the current was turned off. When *V*_GS_ ≤  − 4.5 V, the gate bias of open-gate devices could still effectively modulate the current across a wide range, which is not observed in the normal device. With an increase in *W*_O_, the sample *I*_DS_ could be modulated over a larger *V*_GS_ range. As shown in Fig. 2e, when *V*_GS_ =  − 4 V, there is a kink near the linear-saturation crossover of the output characteristic curve. The specific mechanism of kink effect is still controversial, but it is generally believed that this kink is related to electron traps in AlGaN/GaN heterojunction materials^18–20^. With the increase of *V*_DS_, the channel electrons are first captured by the trap and then escape from the trap, resulting in the channel current first decreasing and then increasing. It should be noted that for the material in this paper, due to the low electron trap density, the kink effect can be obvious only when the channel 2DEG density (*n*_2D_) is low. For open-gate devices, since the open region always maintains a high *n*_2D_, which will be described in detail later, there is no obvious kink effect.

As an important parameter, *V*_th_ can reflect the voltage range in which *V*_GS_ can effectively modulate *I*_DS_. Using the constant current method, when the saturation current reaches the order of 10^−8^ A, the corresponding gate bias is defined as *V*_th_. The values obtained were *V*_th_ =  − 4.5, − 6, − 7, and − 8 V, for the four samples labeled 1, 2, 3, and 4, respectively. This is consistent with what is shown in Fig. 2. It was found that open-gate devices had a lower *V*_th_ than normal devices. As *W*_O_ increased, *V*_th_ changed in the negative direction, so the *I*_DS_ of the samples could be modulated over a larger gate voltage range. In other words, the *V*_th_ of the devices could be modulated simply by changing *W*_O_.

**Figure 3:**
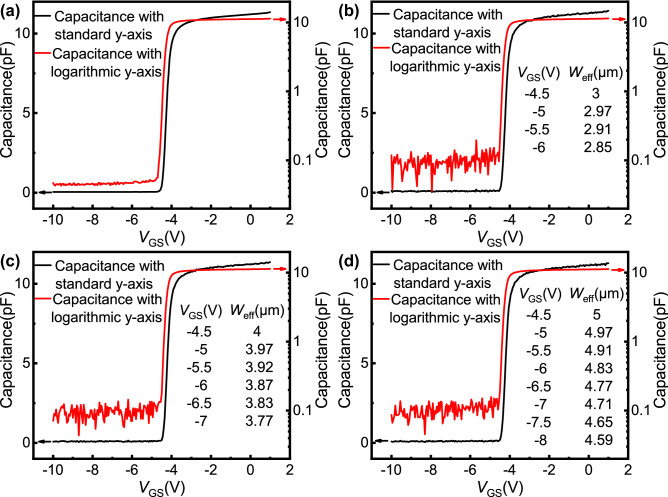
The measured C–V characteristics with standard y-axis and logarithmic y-axis: (**a**) sample 1, (**b**) sample 2, (**c**) sample 3, and (**d**) sample 4. When measuring C–V characteristics, different potentials are applied to gate and source respectively, and source and drain are not shorted. For open-gate devices, the capacitances of the two gates are measured separately and summed to obtain the capacitance shown in the figure. The effective widths of the open region under different gate bias calculated according to the C–V characteristics are also marked in the figure.

The capacitance–voltage (C–V) characteristics with standard y-axis and logarithmic y-axis of four samples are shown in Fig. 3. It can be seen that the C–V curves of the four samples are almost the same when *V*_GS_ >  − 4.5 V. This is because the capacitance of the device obtained from the C–V measurement in this voltage range only corresponds to the region underneath the gate, and the gate areas of the four samples are very close. When the *V*_GS_ is about − 4.5 V, the capacitance of the four samples decreases rapidly to a minimum, which means that the 2DEG underneath the gate region is almost depleted around *V*_GS_ =  − 4.5 V. For the 2DEG in the open region, specific analysis will be carried out as follows.

With the change of gate bias, a lateral electric field may modulate 2DEG in the open region, which is observed in in-plane-gate field effect transistors (IPGFETs)^21,22^. However, for IPGFETs, the barrier layer below the gate is metallized by annealing, so that the gate and channel 2DEG are in the same plane, which further leads to a strong role of the lateral electric field. For AlGaN/GaN open-gate devices, since the gate and channel 2DEG are separated by an AlGaN barrier layer, the lateral electric field cannot be established directly as IPGFETs. However, the gate can still modulate channel 2DEG through the fringe electric field, which is similar to the AlGaAs/GaAs split-gate transistor^23–26^. According to the relevant research results of AlGaAs/GaAs split gate structure^23–25^, before the 2DEG underneath the gate is depleted, the gate bias will not affect the 2DEG in the open region. After that, due to the effect of the fringe electric field, the gate bias will change the amount of charge in the open region, which will be reflected in the C–V characteristics. As shown by the C–V curves with logarithmic y-axis in Fig. 3, it can be seen that when *V*_GS_ ≤  − 4.5 V, although the value is very small, the capacitance of the open-gate device is significantly greater than that of the normal device, and the difference between them reflects the charge consumption in the open region caused by the gate fringe electric field. For the open region, the quantity of electron charge consumed by the fringe electric field under different gate bias can be expressed as

1 $$\Delta Q = \int_{{V_{GS} }}^{ - 4.5} {C_{O} dV} - \int_{{V_{GS} }}^{ - 4.5} {C_{N} dV} .$$

Here, *C*_*O*_ and *C*_*N*_ are the capacitance of the open-gate device and normal device, respectively.

The studies on AlGaAs/GaAs split-gate devices show that the effective width of the open region (*W*_eff_) will narrow due to the consumption of 2DEG electrons by the fringe electric field^23–25^. As for whether the *n*_2D_ of the narrowed open region changes with *V*_GS_, different studies show different results^23,25,26^. For the convenience of calculation, we adopt the results of Thornton et al.^23^ and assume that the *n*_2D_ will remain constant as the *W*_eff_ is reduced. When the fringe electric field consumes few 2DEG electrons in the open region, this assumption is obviously reasonable, and the narrowed *W*_eff_ can be expressed as

2 $$W_{eff} = W_{O} - \Delta Q/en_{2D} L_{G} ,$$

where *e* is the absolute value of the electronic charge.

For open-gate devices, the specific value of *W*_eff_ under different gate bias can be obtained and marked in Fig. 3. It can be seen that the change of *W*_eff_ caused by fringe electric field is very small, which is not enough to pinch off the channel. Therefore, in addition to the modulation of fringe electric field on 2DEG in the open region, there must be a new working mechanism to modulate the current in the open region until it is turned off.

Based on the above analysis, it can be concluded that the open-gate samples have different working modes before and after *V*_GS_ =  − 4.5 V. When *V*_GS_ >  − 4.5 V, the whole channel, including the gate region and the open region, is conductive, and the open-gate device operates in mode 1. At this time, as *V*_GS_ changes negatively, the *n*_2D_ underneath the gate decreases, further leading to the decrease of *I*_DS_, which is the same as the working mechanism of normal devices. When the open-gate device operates in mode 2 (*V*_GS_ ≤  − 4.5 V), the 2DEG underneath the gate region becomes depleted, and only the open region is conductive. In this mode, as *V*_GS_ changes negatively, while the gate fringe electric field reduces *W*_eff_, there is another mechanism affecting the channel electrons, which jointly modulate the channel current. The larger the width of the opening, the greater the initial current of mode 2, and the more difficult it is to turn off. Therefore, *V*_th_ changes negatively with an increase in *W*_O_.

**Figure 4:**
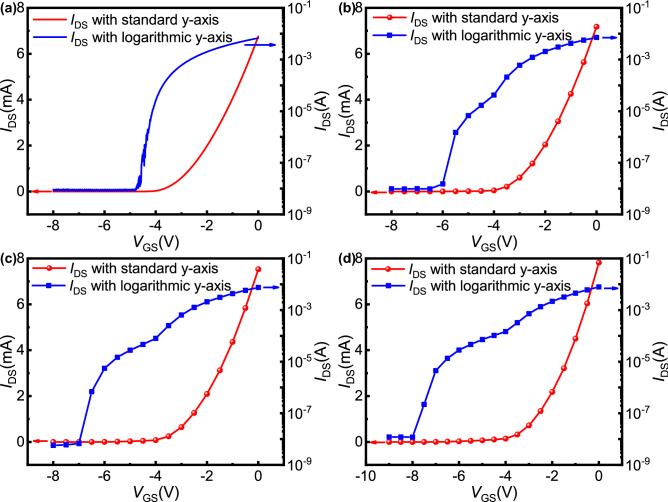
The measured transfer characteristics with standard y-axis and logarithmic y-axis: (**a**) sample 1, (**b**) sample 2, (**c**) sample 3, and (**d**) sample 4. The drain-source voltage is constant at 10 V during measurement. The transfer characteristics of open-gate devices are extracted from their output characteristics.

To compare the modulation ability of the devices in the two working modes, it is necessary to calculate the transconductance before and after *V*_GS_ =  − 4.5 V. Figure 4 shows the transfer characteristics with standard y-axis and logarithmic y-axis of four samples, where the drain-source voltage (${V}_{DS}$) is 10 V. Since the two gates of the open-gate device are not connected, during the measurement of transfer characteristics, when a varying bias is applied to one gate, the same bias needs to be applied to the other gate at the same time, which cannot be achieved due to the instrument limitations. Therefore, we can only find the *I*_DS_ corresponding to different *V*_GS_ from the output characteristics under the condition of *V*_DS_ = 10 V, and extract them to form the transfer characteristics of open-gate devices. It can be clearly seen from Fig. 4 that the turn-off voltages of the four samples are basically consistent with the threshold voltages extracted by constant current method. The turn-off currents of the four samples are all in the order of 10^−8^ A, which indicates that the current can be turned off completely even for the open-gate devices. The transconductance of the devices can be obtained by deriving the transfer characteristic curve. The calculated maximum transconductance (*g*_m,max_) of the open-gate samples before and after *V*_GS_ =  − 4.5 V are shown in Table 1. It can be seen from the table that for open-gate devices, *g*_m,max_ when *V*_GS_ >  − 4.5 V is much larger than that when *V*_GS_ ≤  − 4.5 V, which indicates that the modulation ability of devices is significantly weakened when *V*_GS_ ≤  − 4.5 V.

**Table 1:** The calculated maximum transconductance of samples 2, 3, and 4.

	*g*_m,max_ (S)		
	Sample 2	Sample 3	Sample 4
*V*_GS_ > − 4.5 V	3.09 × 10^−3^	3.38 × 10^−3^	3.56 × 10^−3^
*V*_GS_ ≤ − 4.5 V	3.54 × 10^−5^	5.17 × 10^−5^	7.42 × 10^−5^

**Figure 5:**
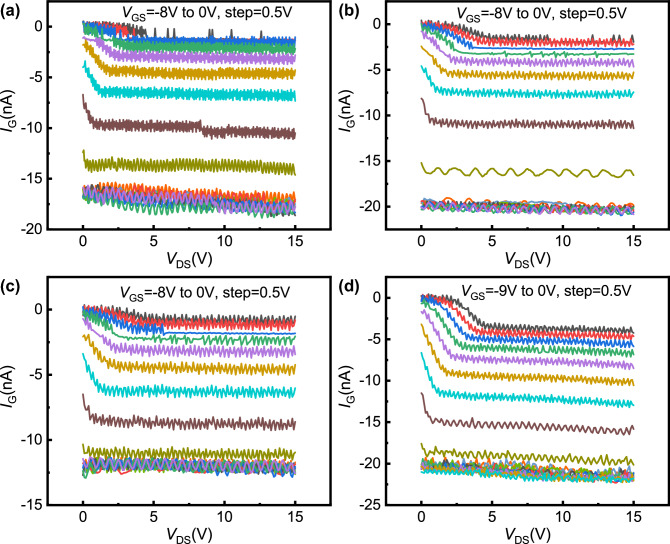
The measured gate leakage current as a function of the gate bias: (**a**) sample 1, (**b**) sample 2, (**c**) sample 3, and (**d**) sample 4.

Figure 5 shows the variation of the measured gate leakage current (*I*_G_) with *V*_GS_ for the four samples. It can be seen that the leakage current of these four samples has little difference. The maximum leakage current value of each sample is in the order of 10^−8^ A, which is in the same order as the turn-off current and far less than the turn-on current. When *V*_GS_ ≤  − 4.5 V, the leakage current of each sample remains almost unchanged. This shows that the effect of *I*_G_ on *I*_DS_ can be ignored when the device is on, and the modulation of gate bias on the current in the open region is not directly caused by *I*_G_. In addition, relevant research shows that the electrons leaking from the gate will be trapped in the empty surface state, thus forming a virtual gate^27^, which may affect the modulation of channel *n*_2D_. However, the virtual gate formed by the above process should be located in the region between the gate and the drain^27^, which is distributed on both sides of the open region rather than directly above it. Therefore, even if the virtual gate caused by gate leakage exists, the *n*_2D_ in the open region cannot be directly modulated.

### Basic working mechanism

We hypothesize that for open-gate devices, the *V*_GS_ modulates the channel current in the open region by changing the electron mobility through the PCF scattering mechanism. In AlGaN/GaN HFETs, there are multiple scattering mechanisms, such as dislocation (DIS) scattering, acoustic phonon (AP) scattering, polar optical phonon (POP) scattering, interface roughness (IFR) scattering, in addition to PCF scattering^28–30^. For a specific region in the channel, the scattering mechanisms other than PCF scattering are only correlated with *V*_GS_ through the *n*_2D_ there. Since the *n*_2D_ in the narrowed open region remains unchanged, it can be considered that the other scattering mechanisms in this region do not change with *V*_GS_. The PCF scattering is not only dependent on the *n*_2D_ in the open region, but also affected by the additional polarization charge density (σ) underneath the gate region of the open-gate device. The polarization charges at the interface of the AlGaN/GaN heterostructure are uniformly distributed before the device is fabricated^31,32^. However, after the device is fabricated, when *V*_GS_ is applied, the strain of the barrier layer underneath the gate changes owing to the existence of the inverse piezoelectric effect^33^. This non-uniform strain will lead to the uneven distribution of polarization charges, thereby generating additional polarization charges and scattering the channel electrons. This is what is known as PCF scattering^34,35^. For open-gate devices, as *V*_GS_ changes negatively, the additional polarization charge underneath the gate will increase, and the PCF scattering to the 2DEG electrons in the open region will increase accordingly. This will lead to a decrease in electron mobility and an increase in resistance in the open region. As a result, the current in the open region decreases as *V*_GS_ changes negatively, so that the gate bias modulates the channel current.

PCF scattering is essentially a Coulomb scattering, which reflects the interaction between additional polarization charges and channel electrons. In the AlGaN/GaN heterostructure, the distribution of channel 2DEG is quantized and can be expressed by a wave function, written as $\Psi \left( {x,y,z} \right) = A^{{ - {1 \mathord{\left/ {\vphantom {1 2}} \right. \kern-\nulldelimiterspace} 2}}} \psi \left( z \right)\exp \left( {ik_{x} x + ik_{y} y} \right)$^36,37^. Here, *A* is the 2-D normalization constant, *k*_*x*_ and *k*_*y*_ are the components of the wave vector ***k*** in the *x* and *y* directions, respectively, $\psi \left( z \right) = \left( {{{b^{3} z^{2} } \mathord{\left/ {\vphantom {{b^{3} z^{2} } 2}} \right. \kern-\nulldelimiterspace} 2}} \right)^{{{1 \mathord{\left/ {\vphantom {1 2}} \right. \kern-\nulldelimiterspace} 2}}} \exp \left( {{{ - bz} \mathord{\left/ {\vphantom {{ - bz} 2}} \right. \kern-\nulldelimiterspace} 2}} \right)$ is the Fang-Howard variational wave function, $b = \left( {{{33m^{ * } e^{2} n_{2D} } \mathord{\left/ {\vphantom {{33m^{ * } e^{2} n_{2D} } {8\varepsilon_{0} \varepsilon_{s} \hbar^{2} }}} \right. \kern-\nulldelimiterspace} {8\varepsilon_{0} \varepsilon_{s} \hbar^{2} }}} \right)^{{{1 \mathord{\left/ {\vphantom {1 3}} \right. \kern-\nulldelimiterspace} 3}}}$ is the variational parameter, m^*^ is the electron effective mass of the GaN material, *ℏ* is the reduced Planck constant, *ε*_0_ is the dielectric permittivity, and *ε*_s_ is the static dielectric constant of GaN.

For samples 2, 3, and 4, taking the open region as a benchmark, meaning that the additional polarization charge in the open region is considered to be zero, only the region underneath the gate has a negative additional polarization charge under negative *V*_GS_. The two gates on both sides of the opening are symmetrical, and the voltages applied to the two gates are the same. Therefore, the additional polarization charges underneath the two gates are equal, expressed as *σ*_G1_ = *σ*_G2_ = *σ*_G_. As mentioned above, when *V*_GS_ ≤  − 4.5 V, only the open region of the device is conductive. At this time, it is only necessary to consider the scattering effect of the additional polarization charges underneath the gate region on the electrons in the open region. This effect can be expressed by the PCF scattering potential, which can be written as^35^

3 $$\begin{aligned} V\left( {x,y,z} \right) & = - \frac{e}{{4\pi \varepsilon_{s} \varepsilon_{0} }}\int_{{ - \frac{{L_{G} }}{2}}}^{{\frac{{L_{G} }}{2}}} {dx^{\prime}} \int_{0}^{{\frac{{W - W_{eff} }}{2}}} {\frac{{\sigma_{G} }}{{\sqrt {\left( {x - x^{\prime}} \right)^{2} + \left( {y - y^{\prime}} \right)^{2} + z^{2} } }}dy^{\prime}} \\ & \quad - \frac{e}{{4\pi \varepsilon_{s} \varepsilon_{0} }}\int_{{ - \frac{{L_{G} }}{2}}}^{{\frac{{L_{G} }}{2}}} {dx^{\prime}} \int_{{\frac{{W + W_{eff} }}{2}}}^{W} {\frac{{\sigma_{G} }}{{\sqrt {\left( {x - x^{\prime}} \right)^{2} + \left( {y - y^{\prime}} \right)^{2} + z^{2} } }}dy^{\prime}} . \\ \end{aligned}$$

The PCF scattering potential interacts with the electrons in the open region and scatters the electrons from the initial state ***k*** to the final state ***k*′**, which can be represented by the matrix element as^35,36^

4 $$\begin{aligned} M_{k \to k^{\prime}} & = A^{ - 1} \int_{0}^{\infty } {\psi_{k^{\prime}}^{ * } \left( z \right)} \\ & \quad \times \left[ {\int_{{ - \frac{{L_{G} }}{2} - L_{GS} }}^{{\frac{{L_{G} }}{2} + L_{GD} }} {dx} \int_{{\frac{{W - W_{eff} }}{2}}}^{{\frac{{W + W_{eff} }}{2}}} {V\left( {x,y,z} \right)\exp \left( { - iq_{x} x - iq_{y} y} \right)dy} } \right]\psi_{k} \left( z \right)dz \\ & = A^{ - 1} \int_{0}^{\infty } {\psi_{k^{\prime}}^{ * } \left( z \right)\left[ {V\left( {q_{x} ,q_{y} ,z} \right)} \right]} \psi_{k} \left( z \right)dz. \\ \end{aligned}$$

Here, *q*_*x*_ and *q*_*y*_ are the components of the wave vector change ***q*** = ***k*′** − ***k*** in the *x* and *y* directions, respectively. The wave vector change ***q*** satisfies $q = \left| {2\left( {2m^{ * } \hbar^{ - 2} E} \right)^{{{1 \mathord{\left/ {\vphantom {1 2}} \right. \kern-\nulldelimiterspace} 2}}} \sin \left( {{\theta \mathord{\left/ {\vphantom {\theta 2}} \right. \kern-\nulldelimiterspace} 2}} \right)} \right|$, where *E* is the 2DEG electron energy, and *θ* is the scattering angle of the 2DEG electron from ***k*** to ***k*′**^35^.

After determining the matrix element, the derivation related to PCF scattering can be obtained by the standardized process (Supplementary Sect. 1).

**Figure 6:**
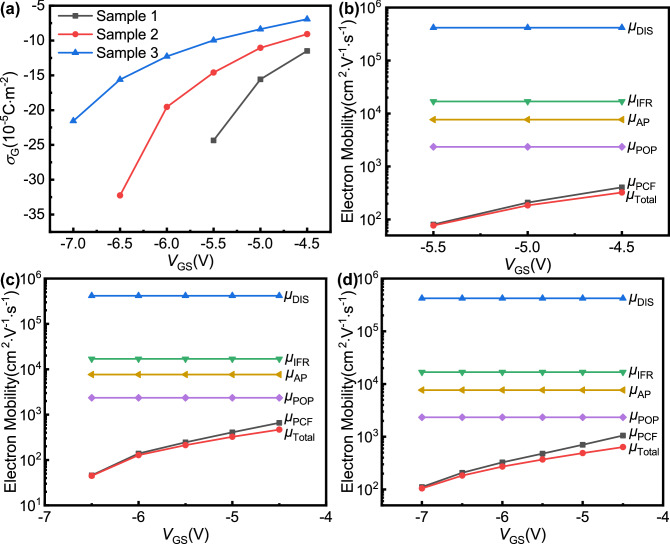
The calculated (**a**) additional polarization charge density for open-gate samples and various electron mobilities for (**b**) sample 2, (**c**) sample 3, and (**d**) sample 4 as a function of the gate bias. *μ*_DIS_, *μ*_IFR_, *μ*_AP_, *μ*_POP_, *μ*_PCF_ and *μ*_Total_ correspond to the electron mobility of DIS scattering, IFR scattering, AP scattering, POP scattering, PCF scattering, and total electron mobility, respectively.

Using the iterative calculation of PCF scattering^38^, the specific values of *σ*_G_ and various electron mobilities can be obtained for different *V*_GS_ values, and the calculated results are shown in Fig. 6. It can be seen that as *V*_GS_ changes negatively, the absolute value of *σ*_G_ increases, which is consistent with the theory of the inverse piezoelectric effect. Under a constant *V*_GS_, larger *W*_O_ values result in smaller absolute values of *σ*_G_. Figure 6 also shows that both *μ*_Total_ and *μ*_PCF_ decrease as *V*_GS_ changes negatively, while *μ*_DIS_, *μ*_AP_, *μ*_POP_, and *μ*_IFR_ do not change with *V*_GS_. As greater negative *V*_GS_ amounts are applied, *μ*_Total_ and *μ*_PCF_ become closer in value. The relationship between electron mobility and *V*_GS_ can be explained as follows. For the narrowed open region of AlGaN/GaN HFETs, *n*_2D_ does not change with *V*_GS_, which means that the scattering mechanisms other than PCF scattering do not change with *V*_GS_ either. As *V*_GS_ changes negatively, the absolute value of *σ*_G_ increases, the PCF scattering becomes stronger, and *μ*_PCF_ decreases accordingly. When other scattering intensities remain unchanged, a decrease in *μ*_PCF_ will inevitably lead to a decrease in *μ*_Total_. On the other hand, when the negative *V*_GS_ value is large, the mobility of PCF scattering is obviously smaller than that of other scattering mechanisms (Fig. 6), which means that PCF scattering occupies a dominant position among the various types of scattering. Therefore, *μ*_Total_ and *μ*_PCF_ are very similar in value. As *V*_GS_ continues to change negatively, the PCF scattering is further strengthened, and its proportion among all scattering mechanisms also increases. Therefore, greater negative *V*_GS_ values result in closer *μ*_Total_ and *μ*_PCF_ values.

When comparing the *μ*_PCF_ of samples 2, 3, and 4, it can also be found that under the same *V*_GS_, smaller opening widths lead to smaller *μ*_PCF_ in the open region. According to previous calculations, it is known that the absolute value of *σ*_G_ will increase as the opening width decreases. On the other hand, when the total width is the same, a smaller open region means a larger gate region. Therefore, as the opening width decreases, the total amount of additional polarization charges underneath the gate region will increase, and the total amount of electrons scattered in the open region will decrease. This will increase the PCF scattering, thereby reducing the *μ*_PCF_ under the same *V*_GS_.

Through the above quantitative calculations, it can be further proven that for open-gate devices, PCF scattering plays an important role. When the 2DEG underneath the gate region is depleted, it is through PCF scattering, which changes the electron mobility in the open region, that *V*_GS_ can modulate *I*_DS_ and further realize the turn-off of the devices. Based on this mechanism, in order to improve the ability of *V*_GS_ to modulate *I*_DS_ in the open region, it is needed to find a way to enhance PCF scattering. Increasing the gate area to increase the additional polarization charge underneath the gate is a feasible method to enhance the PCF scattering, which is also the reason for choosing the large size device in this paper. The above research also shows that the modulation ability of *V*_GS_ to *I*_DS_ in the open region increases with the decrease of opening width. Therefore, if the gate length is reduced and the opening width is reduced in equal proportion, the same mechanism may be expected in devices with shorter gate length. Relevant research is currently in progress.

It has already been established that when *V*_DS_ is applied to AlGaN/GaN HFETs, electrons will be injected onto the surface of the device through the source and trapped by the trap states on the surface, resulting in a drop in the surface potential of the device^39^. In this way, there will be a negative potential difference between the surface and the channel, which is equivalent to a virtual gate with a negative bias being applied to the surface of the device to deplete the 2DEG in the channel. Therefore, the current saturation can be achieved through a mechanism similar to the channel pinch-off for ungated AlGaN/GaN HFETs, and it is also applicable to the open region of open-gate devices. In the above process, the virtual gate is formed by the influence of drain-source electric field, so its potential is not affected by gate bias theoretically. Therefore, this virtual gate can only affect the current saturation, but cannot be used to explain the modulation of gate bias on channel current.

**Figure 7:**
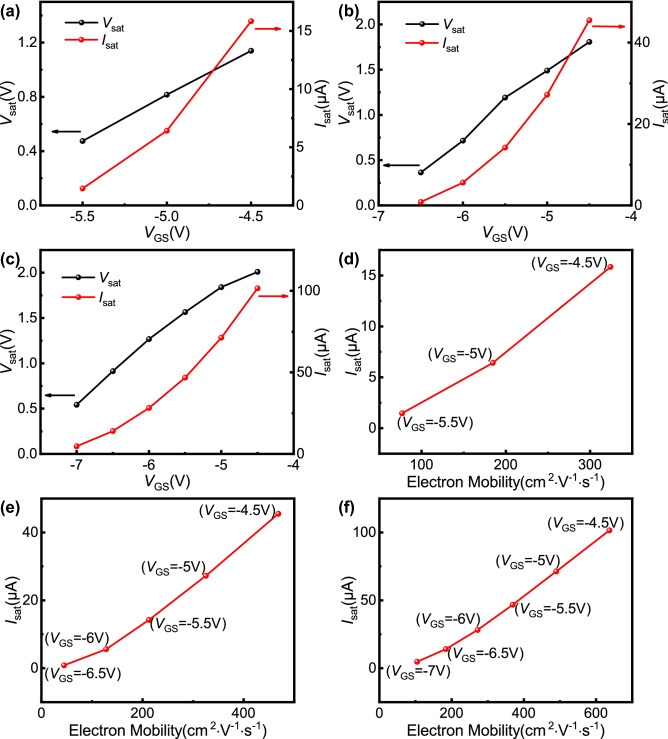
The measured saturation voltage and saturation current as a function of the gate bias for (**a**) sample 2, (**b**) sample 3, and (**c**) sample 4. The measured saturation current as a function of the calculated total electron mobility for (**d**) sample 2, (**e**) sample 3, and (**f**) sample 4. The saturation points are obtained by the method shown in Fig. 2. The electron mobility corresponds to the calculated total electron mobility shown in Fig. 6.

Figure 7a–c display the saturation voltage (*V*_sat_) and saturation current (*I*_sat_) values of samples 2, 3, and 4 as a function of *V*_GS_ when *V*_GS_ ≤  − 4.5 V. *V*_sat_ and *I*_sat_ under different gate biases are obtained by the method shown in Fig. 2. It can be seen that the *V*_sat_ and *I*_sat_ values of each sample decrease as *V*_GS_ changes negatively. According to previous calculations, there is a decrease in electron mobility as *V*_GS_ changes negatively under the influence of PCF scattering, increasing the channel resistance. The conduction part of the device can be regarded as the series connection of the channel region resistance and the ohmic contact resistance, and the ohmic contact resistance does not change with *V*_GS_. As *V*_GS_ changes negatively, the channel resistance increases, while the channel current decreases. This will lead to a reduction in the potential drop on the drain end ohmic contact resistance and further increase the channel potential. In this way, to reach the channel potential corresponding to the saturation point, the *V*_DS_ that needs to be provided will be reduced accordingly. Therefore, *V*_sat_ will decrease as *V*_GS_ changes negatively. Additionally, the channel resistance will increase and *V*_sat_ will decrease, inevitably leading to a decrease in *I*_sat_. When *V*_GS_ ≤  − 4.5 V, the relationships between *I*_sat_ and electron mobility corresponding to different gate biases in the open region of samples 2, 3, and 4 are shown in Fig. 7d–f. It can be seen that *I*_sat_ and electron mobility are approximately linearly related, which is consistent with the paper of Kuzmík et al.^39^ As a result, the working mechanism of the open region can be further proven.

### Application potential

The open-gate device has two conduction modes. When *V*_GS_ ≤  − 4.5 V, only the open region of the device is conductive, and the conduction current is very low. At this time, *V*_GS_ influences the electron mobility through PCF scattering and further modulates *I*_DS_, which has a weaker modulation ability, but can still achieve effective modulation across a larger voltage range. Based on these characteristics of open-gate devices, it is believed that these devices may be suitable for a wide range of low power consumption class-A voltage amplifier applications. Taking sample 4 as an example, it is possible to make theoretical estimates of the performance parameters of the device as a class-A voltage amplifier.

A value of *V*_GS_ =  − 7.5 to − 4.5 V was chosen as the working range of the amplifier, where only the open region of the device was turned on and *V*_GS_ had effective modulation of *I*_DS_, as seen from Fig. 2. The maximum current (*I*_max_) of the device was the saturation current when *V*_GS_ =  − 4.5 V, so *I*_max_ = 1.02 × 10^−4^ A. The quiescent point was selected near the midpoint of the device's operating range so that the device was turned on for the entire period of the input sinusoidal signal. At this time, the amplitude of the input signal voltage (*v*_in_) was 1.5 V. The amplitude of the maximum output voltage (*v*_om_) was determined by the breakdown voltage (*BV*) of the device^40^, which had been tested to be approximately 170 V, so *v*_om_ ≈ *BV*/2 = 85 V. Therefore, as a class-A voltage amplifier, the theoretical maximum value of the device voltage gain (*G*_m_) can be obtained as *G*_m_ = *v*_om_/*v*_in_ ≈ 56.67. In this case, the theoretical value of the direct current power (*P*_DC_) of the device was determined by *I*_max_ and *BV*, expressed as^40^ *P*_DC_ ≈ *I*_max_/2 × *BV*/2 = 4.335 mW ≈ 6.37 dbm. It can be seen that using an open-gate device as a class-A voltage amplifier can effectively amplify a larger input signal with very low power consumption.

As a separate voltage amplifier, the advantages of AlGaN/GaN open-gate HFETs mainly come from the low-transconductance region with large voltage range and small current, which may be replaced by low-transconductance devices with other designs. However, voltage amplifiers usually need to be cascaded with other devices into circuit modules to realize their functions. At this time, the advantages of open-gate devices can be fully reflected. The open-gate device has no special requirements for the structure, doping and thickness of the material, and the preparation process is completely consistent with the normal device, so it can be prepared on the same heterojunction material as the normal device to facilitate circuit integration. If the normal gate structure is adopted, special material structure or process steps are usually required to achieve similar device characteristics, which is not conducive to the integration with normal devices. In addition, open-gate devices have two effective modulation regions: high-transconductance region and low-transconductance region, which is difficult to be realized by other device structures.

Due to their unique characteristics, open-gate devices may have many other applications, which we will continue to study in the future.

## Conclusions

A novel AlGaN/GaN HFET based on open-gate technology was prepared, and a series of experimental tests and theoretical calculations were carried out. For this novel device, when the 2DEG underneath the gate region is depleted, *V*_GS_ can still effectively modulate *I*_DS_. By changing the width of the opening, the modulation of *V*_th_ can easily be achieved. Related calculations have shown that the open-gate device has two working modes with different modulation abilities and working mechanisms. When *V*_GS_ ≤  − 4.5 V, the working mechanism of the open-gate devices are related to PCF scattering and the virtual gate formed by surface electron injection. As a novel device with a new working mechanism, open-gate devices can be used as class-A voltage amplifiers with low power consumption, and also have broad prospects in other applications. Further research in the future will involve trying to reduce the size of the devices.

## Methods

### Sample fabrication

The AlGaN/GaN heterostructure material used in the experiment was grown on a 350 μm SiC substrate. Through metal organic chemical vapor deposition (MOCVD), a 2 μm GaN buffer layer, 1.5 nm AlN insertion layer, 23 nm Al_0.25_Ga_0.75_ N barrier layer, and 2 nm GaN cap layer were grown in sequence. The sheet carrier density of the heterostructure material was about 7.41 × 10^12^ cm^−2^. The device isolation was defined by inductively coupled plasma (ICP) etching. Using electron beam evaporation, Ti/Al/Ni/Au was deposited successively as ohmic contacts, followed by rapid thermal annealing at 850 °C for 30 s in N_2_ atmosphere. The metal used for Schottky contact was Ni/Au, which was also deposited by electron beam evaporation. The patterns of device isolation, ohmic contact, and Schottky contact were all defined by UV-lithography.

### Measurements

The output characteristics of the devices were measured at room temperature by a Keysight B1500A semiconductor parameter analyzer. The C-V characteristics were also measured at room temperature using a Keysight B1520A at 1 MHz.

## Supplementary Information

Supplementary Information.

## Data Availability

The data that support the findings of this study are available from the corresponding author upon reasonable request.
