# Supplementary material for: A novel AlGaN/GaN heterostructure field-effect transistor based on open-gate technology
Source: Sci Rep. 2021 Nov 17;11:22431. doi: 10.1038/s41598-021-01917-9 (PMC8599847; doi:10.1038/s41598-021-01917-9)
Supplement: Supplementary file 1 — Supplementary Information. [file 41598_2021_1917_MOESM1_ESM.pdf]

A novel AlGaN/GaN heterostructure field-effect transistor based on open-gate technology

Yang Liu,[1] Yuanjie Lv,[2] Shuoshuo Guo,[1] Zhengfang Luan,[3] Aijie Cheng,[3] Zhaojun Lin,[1,*] Yongxiong Yang,[1] Guangyuan Jiang,[1] & Yan Zhou[1]

[1]School of Microelectronics, Institute of Novel Semiconductors, Shandong University, Jinan 250101, China.
[2]National Key Laboratory of Application Specific Integrated Circuit (ASIC), Hebei Semiconductor Research Institute, Shijiazhuang 050051, China.
[3]School of Mathematics, Shandong University, Jinan, 250100, China.
[*]email: linzj@sdu.edu.cn

Supplementary Section 1. The standard PCF scattering theoretical model and related formulas after determining the matrix element

Based on the determined matrix element, the energy-dependent scattering rate of PCF scattering can be obtained, and is written as[S1–S4]

$$\frac{1}{\tau_{PCF}(E)} = \frac{Am^*}{2\pi\hbar^3}\int_{-\pi}^{\pi}\left|\frac{M_{k\to k'}}{S(q,T_e)}\right|^2 (1-\cos\theta)\,d\theta, \tag{1}$$

where T_e is the 2DEG electron temperature, and $S(q,T_e)$ represents the screening function, which can be expressed by[S1,S2]

$$S(q,T_e) = 1 + \frac{e^2F(q)\Pi(q,T_e,E)}{2\varepsilon_0\varepsilon_s q}. \tag{2}$$

Here, $F(q)$ is the form factor, written as[S1,S2]

$$F(q) = \int_0^\infty\int_0^\infty \psi^2(z)\psi^2(z')\exp(-q|z-z'|)\,dz dz', \tag{3}$$

and the static polarizability function $\Pi(q,T_e,E)$ can be written as[S1,S2]

$$\Pi(q,T_e,E) = \frac{m^*}{4\pi\hbar^2 k_B T_e}\int_0^\infty \frac{1-\Theta(q-2k_F)\left[1-(2k_F/q)^2\right]^{1/2}}{\cosh^2\left[(E_F-E)/2k_BT_e\right]}dE, \tag{4}$$

where $\Theta(x)$ is the usual step function, $k_F = (2\pi n_{2D})^{1/2}$ is the Fermi wave vector, k_B represents the Boltzmann constant, and E_F is the Fermi energy.
Finally, based on the Fermi statistics, the momentum relaxation time of PCF scattering can be obtained as[S2,S4,S5]

$$\tau_{PCF} = \int \tau_{PCF}(E)E\frac{\partial f_0(E)}{\partial E}dE \Big/ \int E\frac{\partial f_0(E)}{\partial E}dE, \tag{5}$$

where $f_0(E)$ is the Fermi function, which can be expressed as

$$f_0(E) = \frac{1}{\exp\left[(E-E_F)/k_BT_e\right]+1}. \tag{6}$$

In addition to PCF scattering, for the other forms of scattering of 2DEG in the open region, their momentum relaxation time τ_{DIS}, τ_{AP}, τ_{POP}, and τ_{IFR} can be obtained by formulas found in the relevant literature.[S1,S2,S6–S8] After determining the momentum relaxation time, we can calculate the electron mobility of each scattering by[S2,S4,S5]

$$\mu = \frac{e\tau}{m^*}. \tag{7}$$

After that, the total electron mobility in the open region can be calculated according to the Matthiessen rule, which is written as

$$\frac{1}{\mu_{Total}}=\frac{1}{\mu_{PCF}}+\frac{1}{\mu_{DIS}}+\frac{1}{\mu_{AP}}+\frac{1}{\mu_{POP}}+\frac{1}{\mu_{IFR}}. \tag{8}$$

References

S1. Luan, C. et al. Theoretical model of the polarization Coulomb field scattering in strained AlGaN/AlN/GaN heterostructure field-effect transistors. *J. Appl. Phys.* **116**, 044507 (2014).

S2. Gurusinghe, M. N., Davidsson, S. K. & Andersson, T. G. Two-dimensional electron mobility limitation mechanisms in $Al_xGa_{1-x}N$/GaN heterostructures. *Phys. Rev. B* **72**, 045316 (2005).

S3. Stern, F. & Howard, W. E. Properties of Semiconductor Surface Inversion Layers in the Electric Quantum Limit. *Phys. Rev.* **163**, 816–835 (1967).

S4. Jiang, G. et al. Polarization Coulomb field scattering with the electron systems in AlGaN/GaN heterostructure field-effect transistors. *AIP Adv.* **10**, 075212 (2020).

S5. Cui, P. et al. Effect of Different Gate Lengths on Polarization Coulomb Field Scattering Potential in AlGaN/GaN Heterostructure Field-Effect Transistors. *Sci. Rep.* **8**, 9036 (2018).

S6. Yang, M. et al. Effect of Polarization Coulomb Field Scattering on Parasitic Source Access Resistance and Extrinsic Transconductance in AlGaN/GaN Heterostructure FETs. *IEEE Trans. Electron Devices* **63**, 1471–1477 (2016).

S7. Cui, P. et al. Influence of Different Gate Biases and Gate Lengths on Parasitic Source Access Resistance in AlGaN/GaN Heterostructure FETs. *IEEE Trans. Electron Devices* **64**, 1038–1044 (2017).

S8. Gelmont, B. L., Shur, M. & Stroscio, M. Polar optical-phonon scattering in three- and two-dimensional electron gases. *J. Appl. Phys.* **77,** 657–660 (1995).
